# Mineral particles stimulate innate immunity through neutrophil extracellular traps containing HMGB1

**DOI:** 10.1038/s41598-017-16778-4

**Published:** 2017-11-30

**Authors:** Hsin-Hsin Peng, Yu-Ju Liu, David M. Ojcius, Chiou-Mei Lee, Ren-Hao Chen, Pei-Rong Huang, Jan Martel, John D. Young

**Affiliations:** 1 Laboratory of Nanomaterials, Chang Gung University, Gueishan, Taoyuan, 33302 Taiwan; 2 Center for Molecular and Clinical Immunology, Chang Gung University, Gueishan, Taoyuan, 33302 Taiwan; 3 Department of Anesthesiology, Chang Gung Memorial Hospital, Gueishan, Taoyuan, 33305 Taiwan; 4 Laboratory Animal Center, Chang Gung Memorial Hospital, Gueishan, Taoyuan, 33305 Taiwan; 5 Chang Gung Immunology Consortium, Chang Gung Memorial Hospital, Gueishan, Taoyuan, 33305 Taiwan; 6 Department of Biomedical Sciences, University of the Pacific, Arthur Dugoni School of Dentistry, San Francisco, CA 94103 USA; 7 Department of Medical Research and Development, Chang Gung Memorial Hospital, Gueishan, Taoyuan, 33305 Taiwan; 8 Department of Molecular and Cellular Biology, College of Medicine, Chang Gung University, Gueishan, Taoyuan, 33302 Taiwan; 9 Laboratory of Cellular Physiology and Immunology, The Rockefeller University, New York, NY 10021 USA; 10 Biochemical Engineering Research Center, Ming Chi University of Technology, Taishan, New Taipei City 24301 Taiwan

## Abstract

Calcium phosphate-based mineralo-organic particles form spontaneously in the body and may represent precursors of ectopic calcification. We have shown earlier that these particles induce activation of caspase-1 and secretion of IL-1β by macrophages. However, whether the particles may produce other effects on immune cells is unclear. Here, we show that these particles induce the release of neutrophil extracellular traps (NETs) in a size-dependent manner by human neutrophils. Intracellular production of reactive oxygen species is required for particle-induced NET release by neutrophils. NETs contain the high-mobility group protein B1 (HMGB1), a DNA-binding protein capable of inducing secretion of TNF-α by a monocyte/macrophage cell line and primary macrophages. HMGB1 functions as a ligand of Toll-like receptors 2 and 4 on macrophages, leading to activation of the MyD88 pathway and TNF-α production. Furthermore, HMGB1 is critical to activate the particle-induced pro-inflammatory cascade in the peritoneum of mice. These results indicate that mineral particles promote pro-inflammatory responses by engaging neutrophils and macrophages via signaling of danger signals through NETs.

## Introduction

Our previous studies have shown that calcium phosphate particles can form spontaneously and ubiquitously in biological fluids^1–16^. These non-living particles—previously misconstrued as living nanobacteria^17,18^—are involved not only in physiological calcification processes such as bone and tooth formation but also in pathological conditions that include chronic kidney disease, atherosclerosis, and ectopic calcification^19–23^. Owing to their composition and association with protein factors, these mineralo-organic complexes can activate cellular responses when coming into contact with host cells, resulting in particle internalization by host cells and subsequent activation of pro-inflammatory responses^8,10^. Nonetheless, it remains unclear whether the particles could interact with neutrophils, or how the inflammatory response may be propagated.

Neutrophils are innate immune cells that participate in the immune response by engulfing and killing pathogens and by secreting various immune mediators^24,25^. Neutrophil activation by pathogens leads to enhanced NADPH oxidase-derived reactive oxygen species (ROS) production and extracellular release of antimicrobial neutrophil extracellular traps (NETs) consisting of nuclear DNA and cytoplasmic and granular components, such as neutrophil elastase and myeloperoxidase. The resulting web-like structure not only prevents the dissemination of pathogens in the body, but also kills bacteria and other microorganisms with antimicrobial factors^26–31^. NET-forming neutrophils undergo NETosis, a process of programmed cell death distinct from apoptosis and necrosis^28,29,32,33^. NETosis can also be triggered by pro-inflammatory and endogenous stimuli, including platelets, monosodium urate crystals, and auto-antibodies^31,34–38^. Appropriate innate immune defense against pathogens or endogenic danger signals requires interactions and cooperation between neutrophils and macrophages^39–41^. While it is known that these interactions involve NETs^41^, much about the nature of the interactions remains to be elucidated.

High-mobility group protein B1 (HMGB1) is a chromatin-associated, DNA-binding protein that stabilizes nucleosomes and stimulates gene transcription^42,43^. When actively secreted or passively released from stimulated immune cells, HMGB1 also acts as a promoter or inducer of inflammation^44–48^. HMGB1 has been implicated in sepsis, sterile inflammation, autoimmune diseases, and cancer^49^. Moreover, HMGB1 induces monocyte secretion of tumor necrosis factor (TNF), interleukin (IL)-1α, IL-1β, IL-6, IL-8, macrophage inflammatory protein (MIP)-1α, and MIP-1β^50^. These observations suggest that HMGB1 plays a role in numerous immune functions.

We have shown that mineralo-organic particles can activate caspase-1 and induce IL-1β secretion in primed macrophages^10^. Here we report that mineral particles also induce NETosis, leading to the NET-driven activation of bystander macrophages via a pathway involving HMGB1, Toll-like receptors 2 and 4 (TLR2/4), and the adaptor protein, myeloid differentiation primary response gene 88 (MyD88). Taken together, these findings reveal a novel mechanism whereby mineralo-organic particles may amplify the inflammatory response by engaging macrophages after stimulating NET formation by neutrophils.

## Results

### Mineralo-organic particles induce NET release by neutrophils

**Figure 1:**
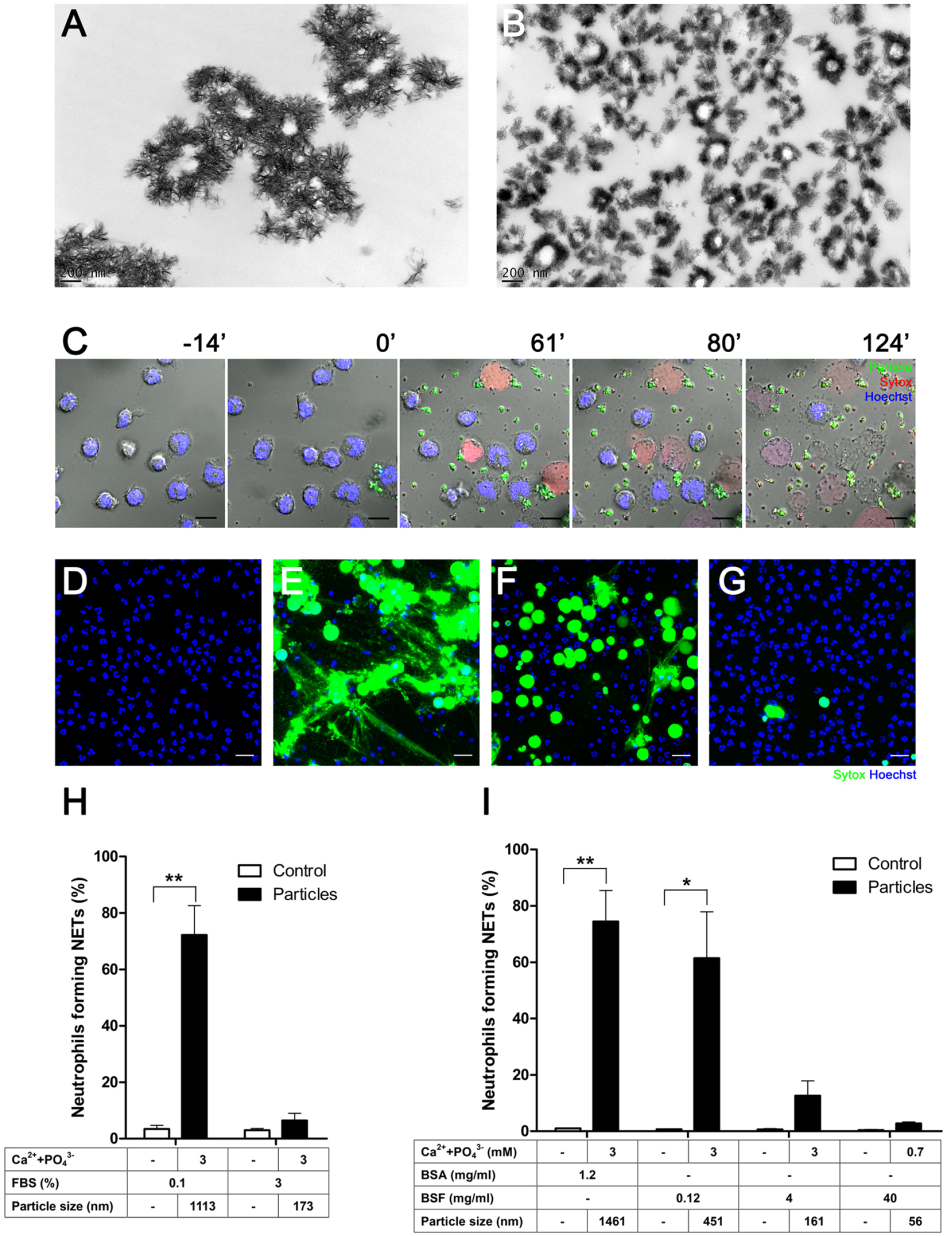
Mineralo-organic particles induce NET release by neutrophils. Mineralo-organic particles were prepared by adding 3 mM of CaCl_2_ and NaH_2_PO_4_ each in DMEM containing (**A**) 0.1% or (**B**) 3% FBS, prior to incubation and preparation for TEM without staining as described in *Methods*. Data are representative of three independent experiments. Scale bars: 200 nm. (**C**) Live cell imaging of human neutrophils stained with Hoechst 33342 (blue) and treated at time 0 with mineral particles (labeled with FITC-bovine serum albumin; FITC-BSA; green). NET-associated DNA was stained with Sytox (red). Data are representative of three independent experiments. Scale bars: 10 μm. Neutrophils were incubated for 2 hours in the absence (**D**) or presence of particles in 0.1% FBS (**E**), 0.12 mg/ml bovine serum fetuin-A (BSF) (**F**), or 40 mg/ml BSF (**G**). NET-associated DNA was stained green by Sytox. Data are representative of three independent experiments. Scale bars: 20 μm. (**H**) Percentage of neutrophils forming NETs 2 hours after addition of particles. Data are shown as means ± standard errors of the mean (SEM) and the results of at least three independent experiments. **p < 0.005, vs. untreated (control) neutrophils. (I) Mineral particles were prepared with BSA or BSF before incubation with neutrophils. Data are shown as means ± SEM and the results of at least three independent experiments. *p < 0.05 and **p < 0.005, vs. untreated (control) neutrophils.

Given that neutrophils represent the first line of defense of the innate immunity, we examined whether these cells respond to mineral particles. We have shown previously that mineralo-organic particles spontaneously form in biological fluids following incubation in cell culture medium^8^. Given the chemical and morphological similarities between these particles and the ones found in the human body, we used the prepared particles to assess the effects of biological particles on innate immune cells^10,13^. Transmission electron microscopy (TEM) observations of mineralo-organic particles revealed pleomorphic morphologies, with elongated, sharp crystals on the surface (Fig. 1A). As previously shown^10^, the size of mineral particles could be controlled by modulating the concentration of serum, with higher fetal bovine serum (FBS) concentrations producing smaller particles (Fig. 1, 0.1% FBS in A vs. 3% FBS in B).

Fluorescein isothiocyanate (FITC)-conjugated bovine serum albumin (BSA)-labeled mineral particles of approximately 1–2 μm—the particle size range shown earlier to elicit pro-inflammatory response in macrophages^10^—were incubated with neutrophils. Cell-impermeable (red, Sytox) and cell-permeable (blue, Hoechst 33342) DNA dyes were used to visualize chromatin at different states of decondensation and extrusion. Live-cell imaging of particle-treated neutrophils revealed NETosis in progress, characterized by disruption of the nuclear and cell plasma membranes, as evidenced by the Sytox signal, accompanied by chromatin decondensation (visualized by the reduced intensity of nuclear DNA signals) and NET release, as shown by the extracellular signal of expelled DNA (Fig. 1C, red, and Supplementary Video S1).

To determine the effects of particle size and morphology on NET formation, we prepared mineral particles in the micro-, sub-micro- and nanometer ranges (Table 1). BSA and bovine serum fetuin-A (BSF), two of the main serum proteins consistently associated with the particles^4^, were used in place of serum during particle preparation (Table 1). Particles in the micrometer range and crystalline particle aggregates elicited intense NET formation within 2 hours of co-culture (Fig. 1E, green, Sytox). For comparison, untreated neutrophils remained largely intact and did not produce NETs (Fig. 1D). Particles in the sub-micrometer range led to moderate NET formation (Fig. 1F), whereas particles in the nanometer range elicited minimal neutrophil response (Fig. 1G). Quantitative analysis indicated that particle-induced NET release is dependent on particle size/crystallinity (Fig. 1H–I). Mineral particles also induced changes in mitochondrial function, as reflected by the loss of mitochondrial membrane potential (red signal) following particle treatment within 30 min of exposure to particles, that eventually led to membrane disruption and chromatin decondensation (Supplementary Fig. S1).

**Table 1:** Conditions for generating mineral particles of various sizes.

Protein concentration	FBS		BSA	BSF		
	0.1%	3%	1.2 mg/ml	0.12 mg/ml	4 mg/ml	40 mg/ml
Ca^2+^ + PO_4_^3-^ (mM)	3					0.7
Size (nm)	1113 ± 43	173 ± 13	1461 ± 67	451 ± 125	161 ± 11	56 ± 3

### NETs induce TNF-α secretion by macrophages

**Figure 2:**
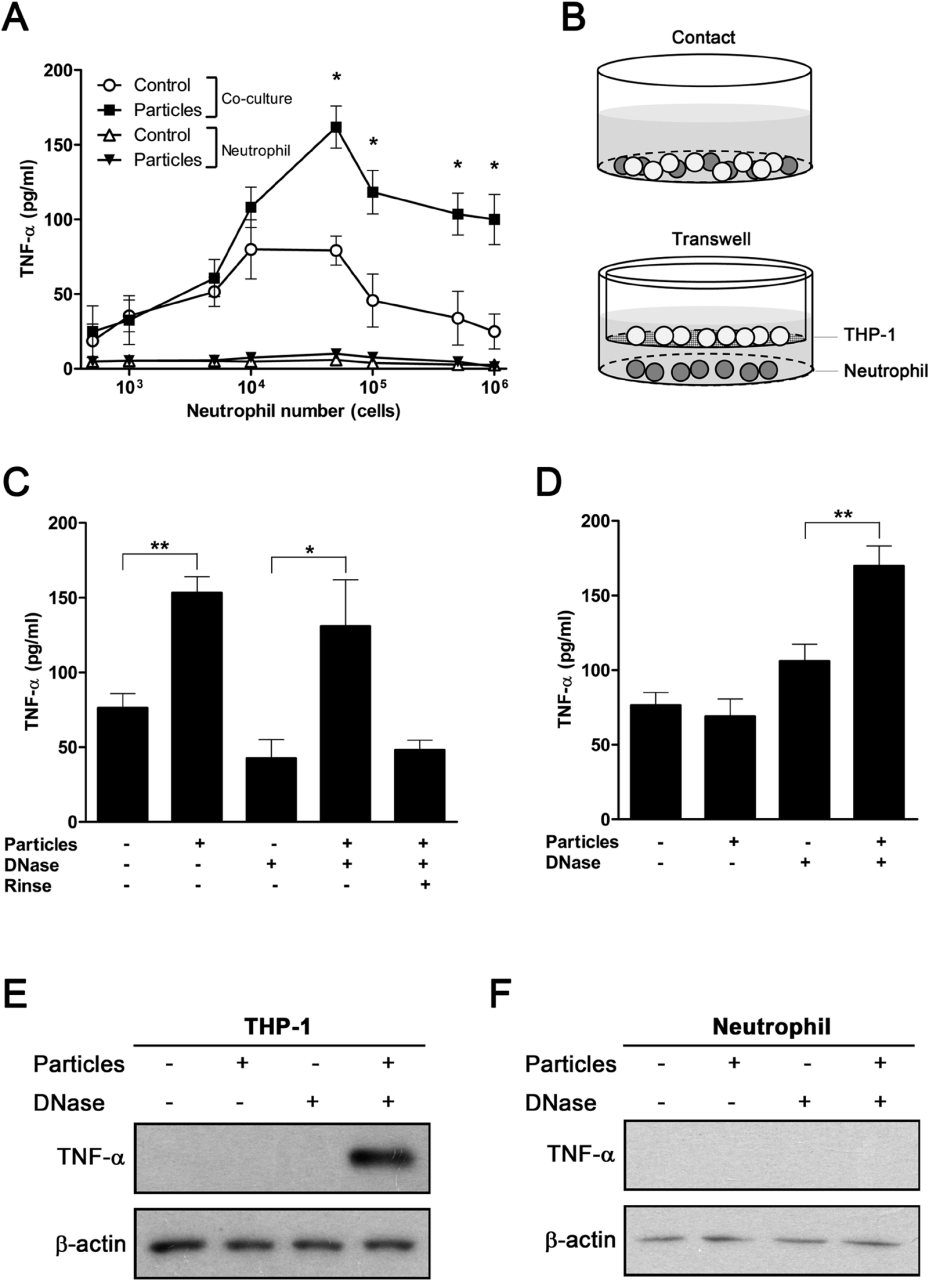
NETs released from particle-treated neutrophils induce secretion of TNF-α by macrophages. (**A**) Human neutrophils were treated with mineralo-organic particles for 30 min, followed by incubation with PMA-primed macrophages for 8 hours. TNF-α secretion was measured using ELISA. Data are shown as means ± SEM and the results of three independent experiments. *p < 0.05, as compared to untreated neutrophils. (**B**) Experimental design used to investigate the neutrophil-macrophage interaction. THP-1 cells were either directly co-cultured with neutrophils (top panel) or grown in the upper chamber of the Transwell system, separated from the neutrophils in the lower chamber by a semi-permeable membrane, which prevented cell-to-cell contact but allowed diffusion of soluble material (bottom panel). (**C**) Co-culture of THP-1 cells with particle-treated neutrophils enhanced TNF-α secretion. Data are shown as means ± SEM and the results of three independent experiments. *p < 0.05 and **p < 0.005, vs. untreated neutrophils. (**D**) Indirect co-culture of THP-1 cells and particle-treated neutrophils in the Transwell system failed to induce TNF-α secretion, but treatment of NETs with DNase enhanced TNF-α secretion. Data are shown as means ± SEM and the results of three independent experiments. **p < 0.005, vs. untreated neutrophils. (**E**) THP-1 cells or (**F**) neutrophils in the Transwell system were subjected to immunoblotting to detect intracellular TNF-α. Data are representative of three independent experiments.

We examined whether particle-stimulated neutrophils activated macrophages via formation of NETs. Untreated and particle-treated neutrophils were co-cultured with phorbol 12-myristate 13-acetate (PMA)-primed THP-1 for 8 hours, and TNF-α secretion from neutrophil-macrophage cultures was measured. Contact co-culture of PMA-primed macrophages and particle-treated neutrophils resulted in induced TNF-α secretion, which reached a maximum at a macrophage neutrophil ratio of 20:1 (Fig. 2A). The induction requires both primed macrophages as well as neutrophil activation by particle exposure, as shown by the lack of significant induction of TNF-α secretion in the absence of macrophages (Fig. 2A). Although PMA induces NETosis in neutrophils^26,28^, these cells do not respond to PMA-primed macrophages in the absence of particles (Fig. 2A). In addition, PMA-primed macrophages alone do not respond to particle stimuli, as shown by the unchanged TNF-α production (Supplementary Fig. S2). Thus, TNF-α production induced by mineral particles requires the presence of both macrophages and neutrophils.

To investigate whether direct contact between neutrophils and macrophages is required for particle-induced TNF-α secretion, we used a Transwell system that prevents cell-to-cell contact but allows diffusion of soluble factors between chambers (Fig. 2B, membrane with 0.4-μm pores). TNF-α secretion was significantly induced upon direct contact between particle-treated neutrophils and macrophages (Fig. 2C). In contrast, neutrophil-macrophage co-culture did not lead to significant alterations in TNF-α secretion in the non-contact Transwell system (Fig. 2D). To determine whether NET-bound factors are responsible for contact induction of immune cells by NETs, we treated the cell culture with DNase I to release DNA-bound mediators. DNase treatment and subsequent removal of DNase-solubilized materials abrogated contact-induced TNF-α production (Fig. 2C). Furthermore, in the Transwell system, DNase treatment enhanced TNF-α secretion (Fig. 2D). These findings confirmed that NETs mediate contact activation by presenting factors that are bound to their nucleic acid structure.

We investigated which cell type is responsible for the secretion of TNF-α in response to particle treatment. Intracellular TNF-α levels were determined in both PMA-primed macrophages and neutrophils in the non-contact Transwell culture system. After DNase was added in the lower chamber, significant intracellular TNF-α was detected in macrophages, whereas no intracellular TNF-α was detected in neutrophils (Fig. 2E,F). Taken together, these results indicate that mineral particles are capable of driving NET release by neutrophils, and that the resulting NETs induce TNF-α secretion by macrophages.

### Mineralo-organic particles induce NET formation in a ROS-dependent manner

**Figure 3:**
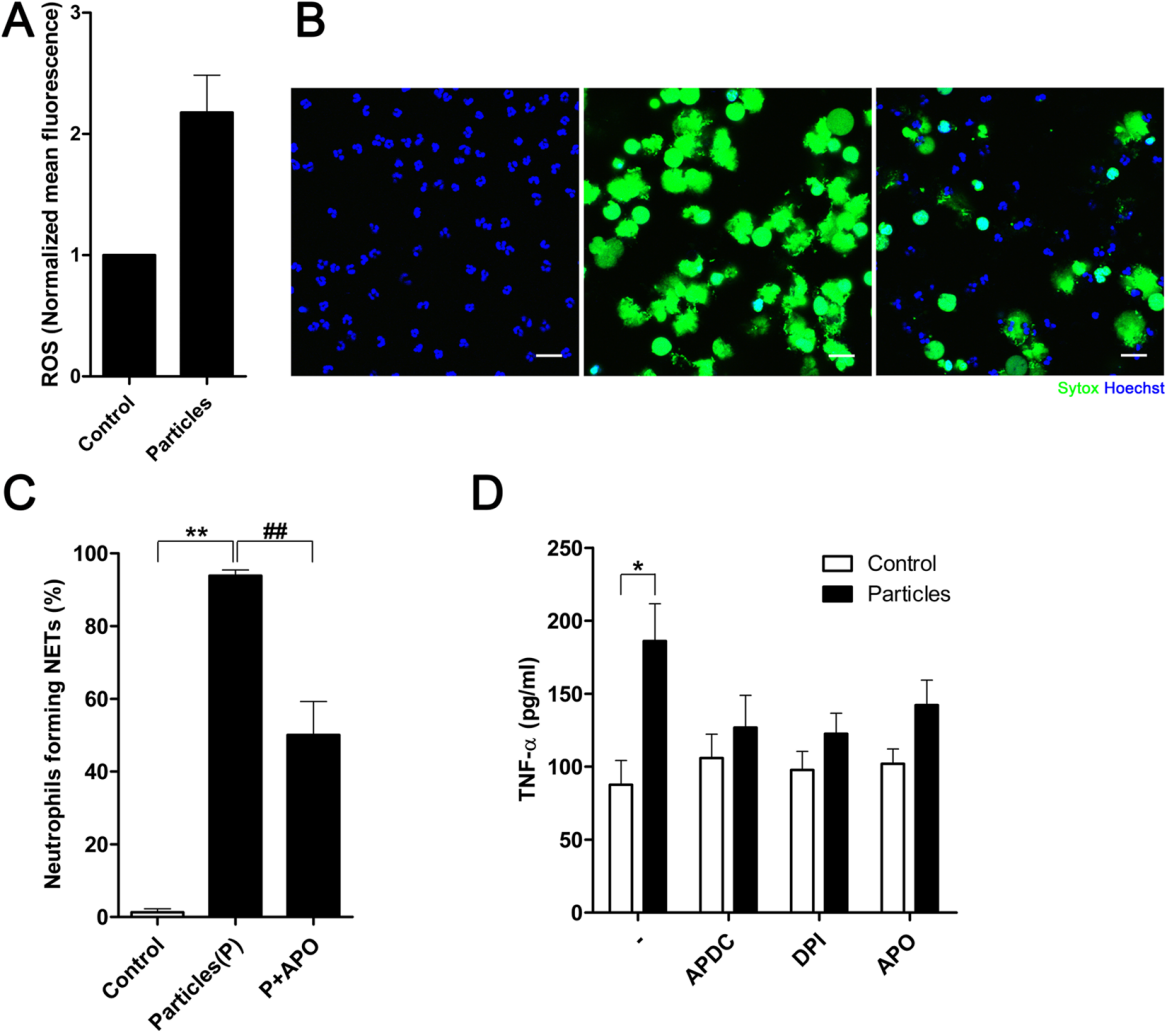
ROS production is critical for particle-induced pro-inflammatory activation. (**A**) ROS production was determined by loading neutrophils with CM-H_2_DCFDA and subjecting the cells to flow cytometry after co-culture with particles. Treatment with mineral particles enhanced intracellular ROS production. Data are shown as means ± SEM and the results of at least three independent experiments. (**B**) Neutrophils were incubated for 2 hours in the absence (left panel) or presence (middle panel) of particles in 0.1% FBS. In addition, concurrent treatment with apocynin (APO) was also carried out in the presence of particles in 0.1% FBS (right panel). NET-associated DNA was stained green by Sytox. Data are representative of three independent experiments. Scale bars: 20 μm. (**C**) Proportion of neutrophils forming NETs in the presence of particles with or without APO in panel (B) was quantified as percentage of total neutrophils. Data are shown as means ± SEM and the results of at least three independent experiments. **p < 0.005, vs. untreated neutrophils. ^##^ *p* < 0.005, vs. particle-stimulated neutrophils. (**D**) TNF-α production by NET-stimulated macrophages was determined by ELISA. Data are shown as means ± SEM and the results of at least three independent experiments. *p < 0.05, vs. untreated neutrophils.

As ROS have been shown to promote NETosis and NET formation in activated neutrophils^28,33^, we investigated whether mineral particles induce ROS production in neutrophils. We observed that ROS production within neutrophils was enhanced upon contact with mineral particles (Fig. 3A).

To investigate whether particle-induced ROS was involved in particle-driven NET formation, we utilized inhibitors targeting ROS production. When treated with apocynin (APO), an NADPH oxidase inhibitor, the NET formation in response to particle stimuli was suppressed (Fig. 3B). Quantitation of the extent of suppression revealed significant reduction of the number of neutrophils forming NETs in response to stimulation with particles (Fig. 3C). Moreover, inhibition of ROS production in neutrophils by NADPH oxidase inhibitors including (2R,4R)-4-aminopyrrolidine-2,4-dicarboxylic acid (APDC), diphenylene iodonium (DPI), and APO also reduced particle-induced TNF-α secretion by NET-stimulated macrophages (Fig. 3D). In short, ROS production in response to particle stimuli is critical for particle-driven neutrophil activation and subsequent NET generation.

### HMGB1 is detected on NETs released from particle-activated neutrophils

**Figure 4:**
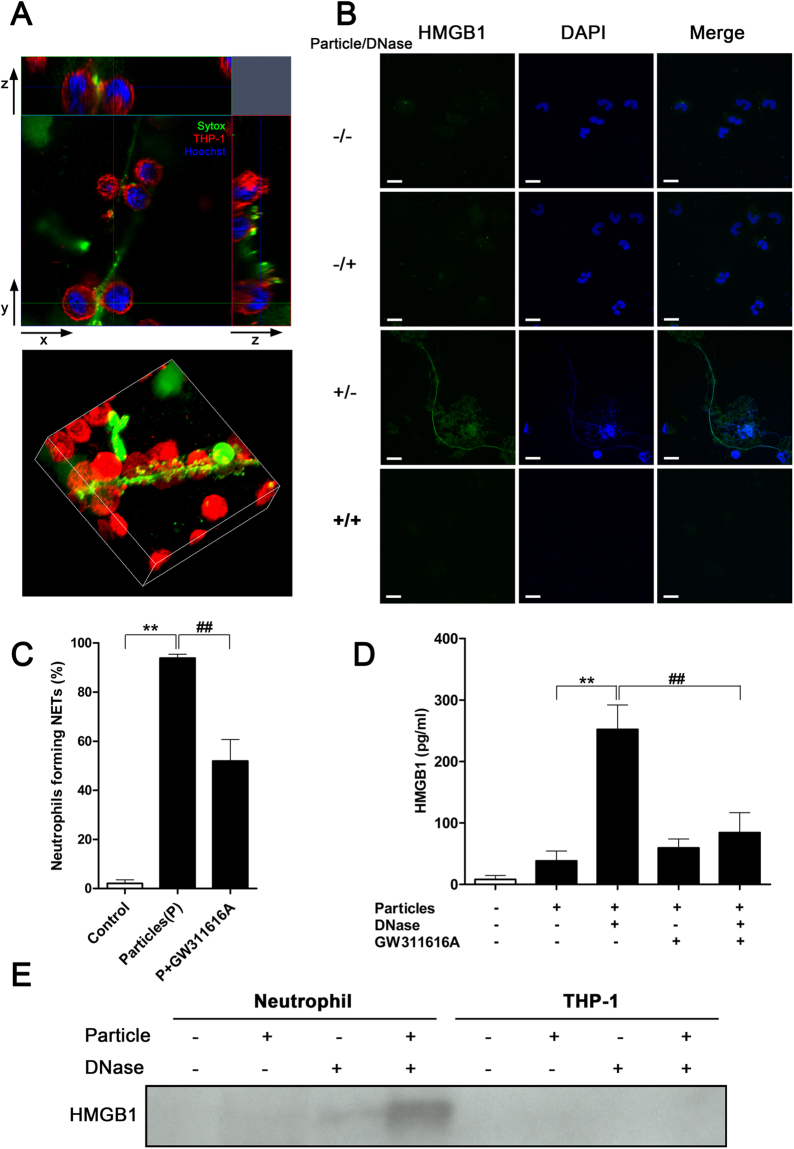
HMGB1 is associated with NETs. (**A**) Cross-section (top panel) and 3D-reconstruction (bottom panel) of NETs interacting with THP-1 cells. NETs were stained green; chromatin DNA, blue. Plasma membrane of THP-1 cells was stained red to outline the cells. THP-1 macrophages closely interacted with NETs. Data are representative of three independent experiments. (**B**) NET-associated HMGB1 was released along with NETs after addition of mineral particles, and digestion of NETs removed the presence of HMGB1. Data are representative of three independent experiments. (**C**) Percentage of neutrophils forming NETs in response to particle stimuli in presence or absence of the neutrophil elastase inhibitor GW311616A for 2 hours. Data are shown as means ± SEM and the results of at least three independent experiments. **p < 0.005, vs. resting (control) neutrophils. ^##^ *p* < 0.005, as compared to particle-stimulated neutrophils. (**D**) Detection of freed NET-bound HMGB1 in culture medium by ELISA after DNase treatment. Data are shown as means ± SEM and the results of at least three independent experiments. **p < 0.005, vs. particle-stimulated, non-DNase-treated neutrophils without GW311616A treatment. ^##^ *p* < 0.005, vs. particle-stimulated, DNase-treated neutrophils without GW311616A treatment. (**E**) Proteins in culture supernatant of particle-treated neutrophils or THP-1 cells were precipitated and subjected to immunoblotting. HMGB1 was detected only in culture medium of particle-treated neutrophils upon DNase treatment to solubilize extracellular NETs. Data are representative of three independent experiments.

We sought to identify and characterize the factor(s) associated with particle-activated NETs that could induce TNF-α production by macrophages. At a macrophage:neutrophil ratio of 20:1, NETs become actively entangled with macrophages (Fig. 4A and Supplementary Video S2), facilitating direct interaction between macrophages and NET-bound molecules. To assess the role of NET-bound HMGB1 in NET-mediated activation of macrophages, we performed immunofluorescence staining on resting and particle-treated neutrophils. In particle-treated neutrophils, HMGB1 was detected in association with NETs, which were stained with 4′,6-diamidino-2-phenylindole (DAPI), whereas extracellular HMGB1 signal was not detected in the untreated neutrophils (Fig. 4B). Staining of NET-bound HMGB1 disappeared after digestion of extracellular DNA by DNase (Fig. 4B).

Treatment with a neutrophil elastase inhibitor (GW311616A) significantly reduced particle-induced NET production (Fig. 4C). Furthermore, inhibition of elastase reduced NET-mediated presentation of HMGB1, as shown by the reduction of free HMGB1 in neutrophils treated with particles and DNase (Fig. 4D). The observed reduction of DNA-bound HMGB1 after treatment with the elastase inhibitor was consistent with the inhibition of NET formation in the absence of elastase activity (Fig. 4D).

The presence of HMGB1 was also assessed by immunoblotting. HMGB1 was prominently detected in the culture supernatant of DNase-treated, particle-activated neutrophils (Fig. 4E), confirming our observations that HMGB1 is bound to NETs. In contrast, HMGB1 was undetectable in the supernatant of macrophage cell culture, with or without particles and in the presence or absence of DNase (Fig. 4E), thus excluding macrophages as a possible source of HMGB1.

### HMGB1 is required for NET-mediated macrophage activation

**Figure 5:**
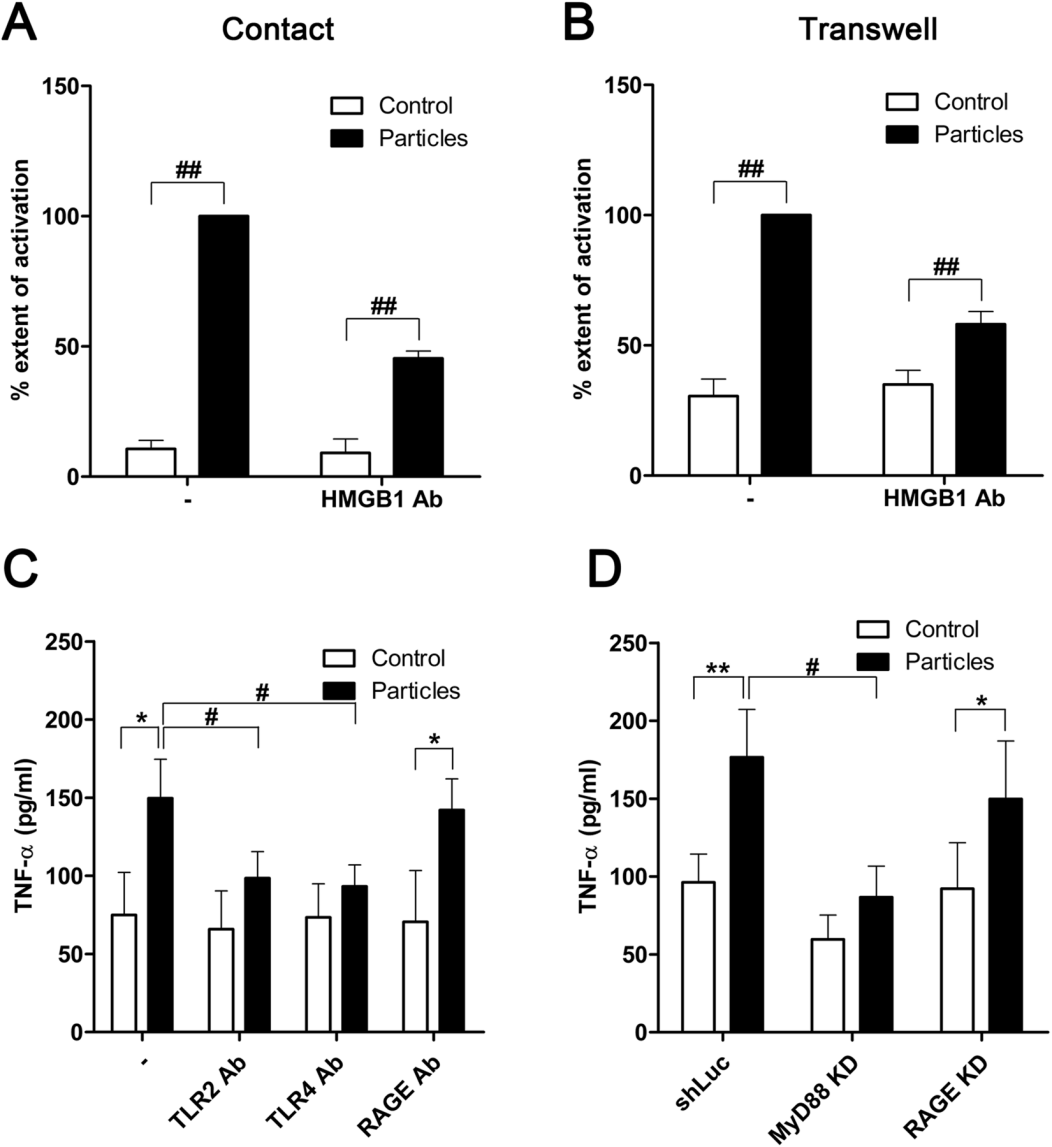
HMGB1 induces macrophage activation via the MyD88-TLR2/4 pathway. (**A**) THP-1 macrophages were co-cultured with particle-treated neutrophils in direct contact in the presence or absence of HMGB1-neutralizing antibodies (HMGB1 Ab). “% extent of activation” represents the extent of activation as indicated by TNF-α release, relative to the full response to particle induction, expressed in percentage (%). Data are shown as means ± SEM for the results of at least three independent experiments. ^##^ *p* < 0.005, vs. particle-stimulated neutrophils. (**B**) Partial inhibition of TNF-α production by anti-HMGB1 antibodies was observed for THP-1 cells in a non-contact system and exposed to DNase-digested NETs. Data are shown as means ± SEM and the results of at least three independent experiments. ^##^ *p* < 0.005, vs. particle-stimulated neutrophils. (**C**) Neutralizing antibodies targeting TLR2, TLR4, or receptor for advanced glycation end products (RAGE) were added to the co-culture system. Data are shown as means ± SEM and the results of at least three independent experiments. *p < 0.05, vs. resting (control) neutrophils. ^#^ *p* < 0.05, vs. particle-stimulated neutrophils. (**D**) THP-1 cells with expression of MyD88 (MyD88 KD) or RAGE stably knocked down (RAGE KD) by shRNA were subjected to the direct co-culture system. Data are shown as means ± SEM and the results of at least three independent experiments. *p < 0.05 and **p < 0.005, vs. resting (control) neutrophils. ^#^ *p* < 0.05, vs. particle-stimulated neutrophils.

To establish the role of NET-associated HMGB1 in driving the particle-induced TNF-α production by macrophages, we neutralized the activity of extracellular HMGB1 using antibodies. Treatment of particle-activated neutrophils with anti-HMGB1 antibodies reduced TNF-α release from bystander macrophages (Fig. 5A). In the Transwell neutrophil-macrophage culture system, TNF-α release by macrophages was reduced when NET digestion by DNase was performed in the presence of HMGB1-neutralizing antibodies (Fig. 5B). HMGB1 therefore is critical for NET-mediated macrophage activation.

### HMGB1 induces macrophage activation via the TLR2/4-MyD88 pathway

To elucidate the mechanism by which HMGB1 mediates macrophage activation in the presence of particle-stimulated neutrophils, we used neutralizing antibodies against mediators of HMGB1 signaling in macrophages. TLR2, TLR4, and the receptor for advanced glycation end products (RAGE) have been reported to mediate HMGB1-induced cellular activation^49,51–56^. Notably, NET-induced TNF-α production was abolished by neutralizing antibodies against TLR2 and TLR4, but antibodies against RAGE showed a limited effect on TNF-α release (Fig. 5C). The adaptor protein, MyD88, is essential for induction of inflammation triggered by all the TLRs except TLR3^57^. We therefore generated a stable MyD88-knockdown (MyD88 KD) THP-1 cell line by using short-hairpin RNA (shRNA), and measured its TNF-α production in response to particle-treated neutrophils. RAGE-knockdown (RAGE KD) THP-1 cells were examined in parallel. Compared with control THP-1 cells expressing shRNA directed against luciferase (shLuc), there was little TNF-α secretion when MyD88 KD THP-1 cells were co-cultured with particle-activated neutrophils (Fig. 5D). In contrast, TNF-α production was not affected by RAGE depletion (Fig. 5D), consistent with the observations made with RAGE-neutralizing antibodies (Fig. 5C). Thus, HMGB1 from particle-induced NETs act specifically via the TLR2/4-MyD88 signaling pathways in macrophages, leading to the secretion of TNF-α.

### Particles induce primary macrophage activation via HMGB1

**Figure 6:**
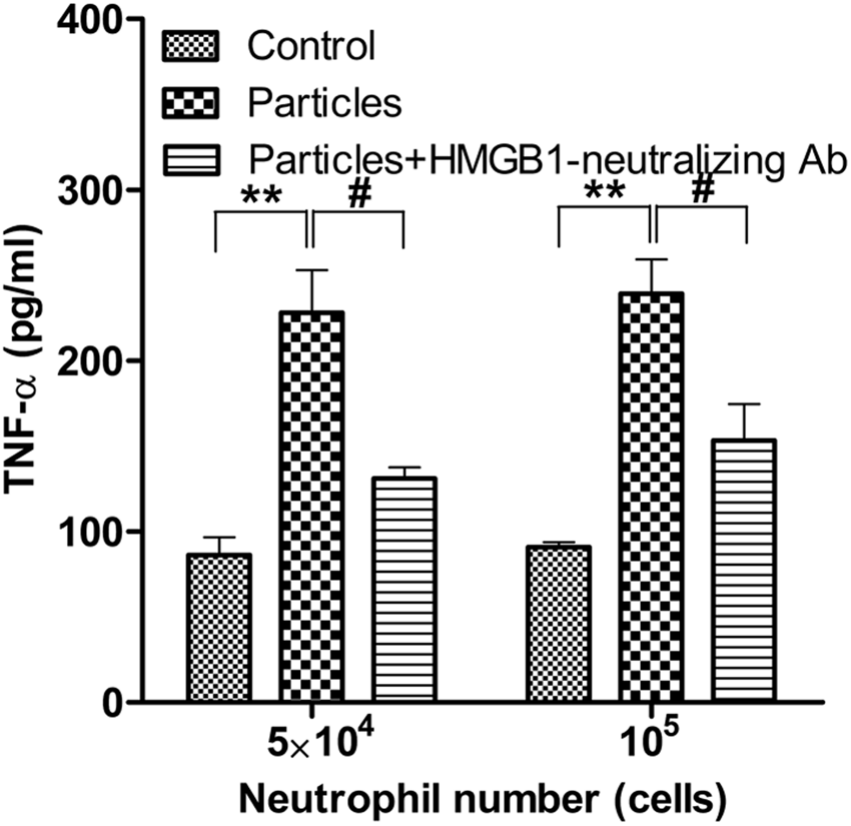
Neutralization of HMGB1 inhibits primary macrophage activation by particle-induced neutrophils. Primary macrophages were co-cultured with particle-treated neutrophils in the presence or absence of HMGB1-neutralizing antibodies. Both neutrophils and macrophages were isolated from the blood of healthy donors. Macrophages (10^6^) were co-cultured with neutrophils at the indicated number and were pre-activated or not by particles. Data are shown as means ± SEM and the results of at least three independent experiments. **p < 0.005, vs. resting (control) neutrophils. ^#^ *p* < 0.05, vs. particle-stimulated neutrophils.

To confirm that the observations made with THP-1 cells were applicable to primary macrophages, we tested macrophages differentiated from the blood of healthy donors. When exposed to particles, neutrophils could induce the pro-inflammatory activity of primary macrophages, as reflected by the increased release of TNF-α (Fig. 6). Treatment with HMGB1-neutralizing antibody effectively abrogated neutrophil-induced macrophage activity (Fig. 6). These results were consistent with our initial findings with the THP-1 cell line for the role of HMGB1 as the mediator of pro-inflammatory signal transmission from activated neutrophil to macrophages.

### Mineralo-organic particles facilitate intraperitoneal inflammatory cascade in mice via HMGB1

**Figure 7:**
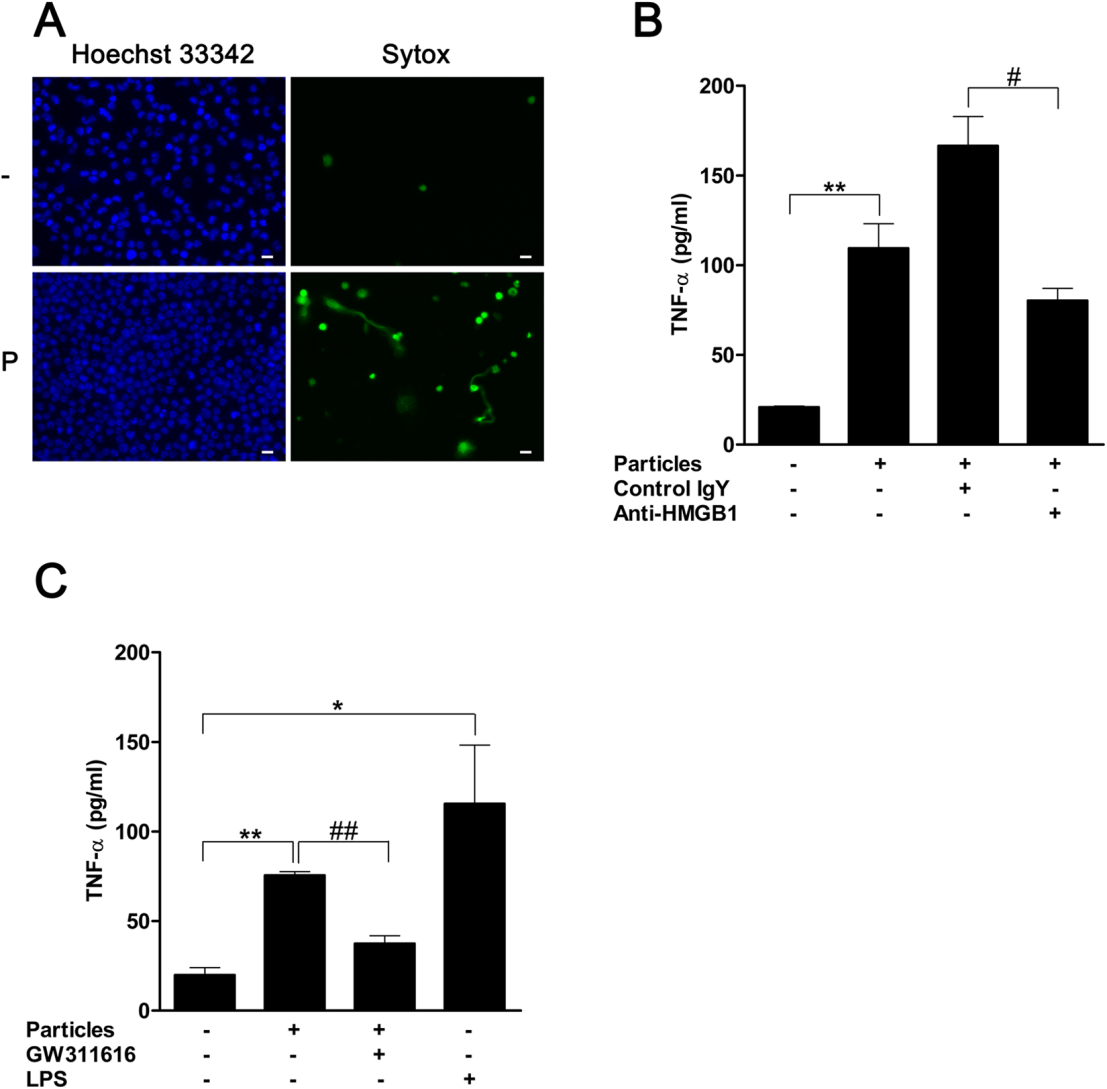
Intraperitoneal mineralo-organic particles promote pro-inflammatory activities in C57BL/6 mice. (**A**) DMEM without (“−”) or with mineralo-organic particles (“P”) were injected into the peritoneum of C57BL/6 mice. Eight hours after injection, intraperitoneal lavage was performed on the treated animals to access intraperitoneal cell population profiles. Data are representative of three independent experiments. Scale bars: 10 μm. (**B**) Mineralo-organic particles were intraperitoneally injected into C57BL/6 mice either together with anti-HMGB1-neutralizing antibodies (“Anti-HMGB1”) or isotype control antibody of chicken IgY (“Control IgY”). A vehicle control without the addition of mineralo-organic particles and antibodies also was included in this study. Two hours after injection of mineralo-organic particles, the RAW264.7 cells previously primed with IFN-γ were intraperitoneally injected into the mice. The inflammatory response elicited after six hours post-injection of mineralo-organic particles was assessed by detecting TNF-α via ELISA. Data are shown as means ± SEM and the results of at least three independent experiments. **p < 0.005, vs. vehicle control. ^#^ *p* < 0.05, vs. Control IgY. (**C**) Particles were intraperitoneally injected into C57BL/6 mice alone or together with GW311616, followed by injection of interferon (IFN)-γ-primed RAW264.7 cells two hours later. The inflammatory response elicited after 6-hour post-injection of particles was assessed by ELISA. Data are shown as means ± SEM and the results of at least three independent experiments. *p < 0.05 and **p < 0.001, vs. vehicle control. ^##^ *p* < 0.005, vs. GW311616-free control.

To confirm particle activity on immune cell components *in vivo*, we injected mineralo-organic particles into the peritoneal cavity of wild type C57BL/6 mice. The peritoneal presence of mineralo-organic particles led to infiltration of mononuclear and polymorphonuclear cells in the peritoneum, as shown by the increased number of cells in the peritoneal lavage (Fig. 7A, lower left panel). The increased cell infiltration occurred in conjunction with heightened activity of NETosis or NET production, which was apparent when staining with Sytox (Fig. 7A, lower right panel).

We examined pro-inflammatory activation by mineralo-organic particles in the peritoneal cavity. To investigate the early post-NETosis downstream consequences before the emergence of any secondary responses, we intraperitoneally injected RAW264.7 cells, a macrophage cell line, to amplify pro-inflammatory responses that might take place within 6 hours after particle injection. The exogenous RAW264.7 cells enabled amplification and detection of increased TNF-α production in response to inflammation induction by treatment with mineral particles (Fig. 7B). Furthermore, when HMGB1-neutralizing antibodies were injected into the peritoneum of animals already exposed to particle stimuli, the particle-induced TNF-α increase was abrogated (Fig. 7B, compare IgY to anti-HMGB1). Consistent with the roles of NETs in facilitating pro-inflammatory responses to particle stimuli, inhibition of NETosis by GW311616 abrogated particle-induced release of TNF-α (Fig. 7C). These results demonstrate that the mineralo-organic particles can elicit pro-inflammatory responses *in vivo* via the action of HMGB1, which was likely presented by NETs in the early stages of the host response to particle stimuli.

## Discussion

**Figure 8:**
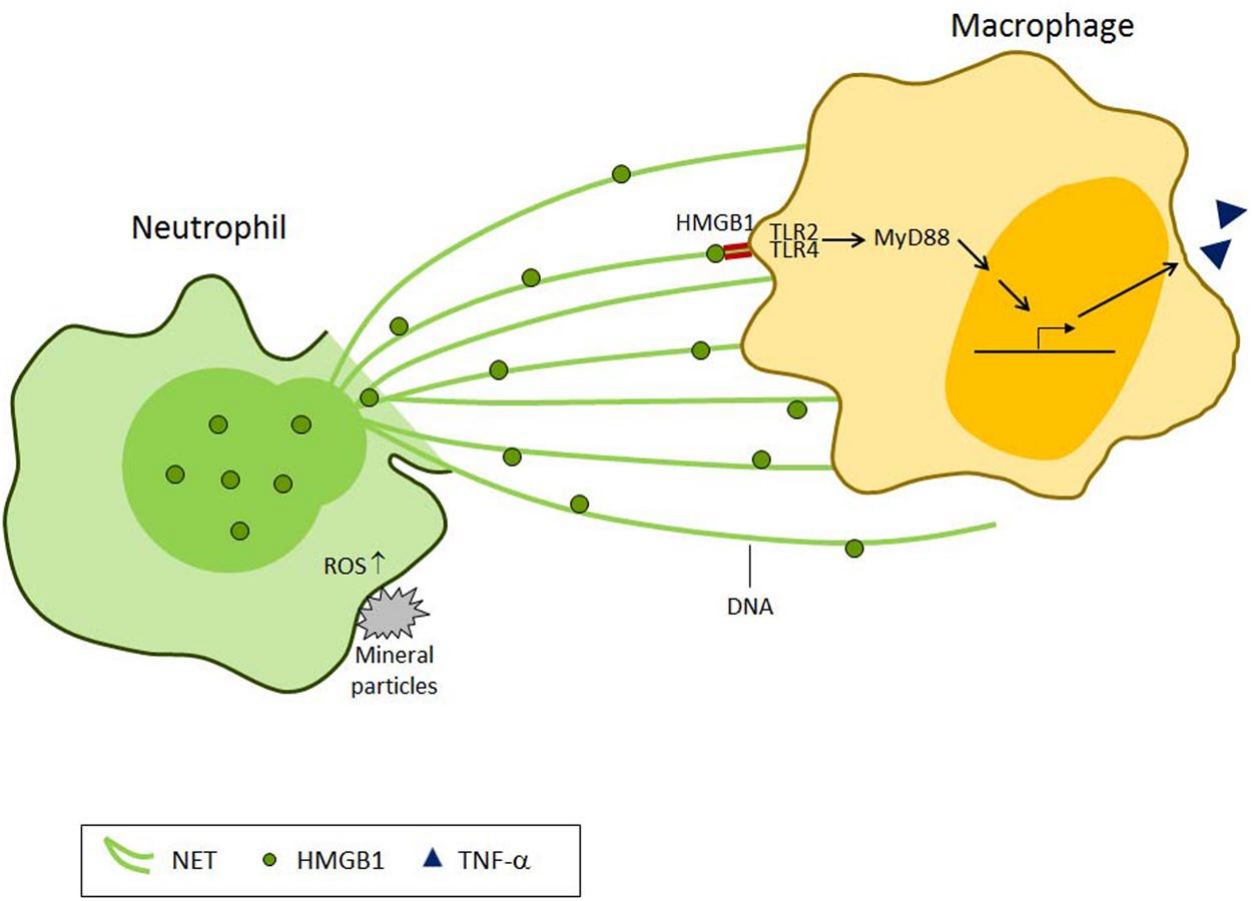
Working model of neutrophil-macrophage crosstalk via NET-bound HMGB1 during activation of neutrophils by mineralo-organic particles. When exposed to mineral particles, neutrophils interact with the particulate matters, which in turn lead to ROS production and NETosis. The released NETs deliver danger signals to bystander macrophages via NET-bound HMGB1. The NET-bound HMGB1 signals via a TLR2/TLR4-dependent pathway and initiates pro-inflammatory signaling cascades in the macrophages, resulting in inflammation via secretion of TNF-α.

The observation that mineralo-organic particles can form in the human body has important implications for understanding the formation of bones and teeth and also for the treatment of human diseases such as atherosclerosis, chronic kidney disease, and ectopic calcification. Exposure to micro- and nanoparticles may be associated with pathological conditions, especially in diseases in which inflammation is a distinguishing feature^38,58^. We have shown in a previous study^10^ that mineralo-organic particles induce the secretion of the pro-inflammatory cytokine IL-1β by macrophages. In the present study, we demonstrate that mineral particles activate NET release by neutrophils, which in turn induce the secretion of TNF-α by macrophages. We therefore propose (as illustrated in Fig. 8) that activation of neutrophils by mineral particles leads to release of NETs containing the danger signal HMGB1, which in turn activates bystander macrophages. These findings suggest that mineralo-organic particles may promote inflammation through NET-associated HMGB1, a mechanism that has not been described previously.

Our results show that, for the particle sizes tested in our study, particles of small sizes (<100 nm) do not induce NETosis, whereas a high level of NET production is elicited by particles of a few micrometers (1–2 μm). These results suggest that mineral particles in the micrometer range may induce NET production by exhausting the phagocytic capacity of neutrophils. Consistent with this possibility, Branzk and colleagues demonstrated that elastase trafficking downstream of ROS production may determine the status of NET release in response to microbes of varying sizes: when phagocytosis overwhelms the responding neutrophils, elastase is directed into the nucleus to initiate NETosis^59^. Our findings are also consistent with previous studies that reported that mineral particles of a few nanometers (1–2 μm) induced pro-inflammatory reactions in monocytes and macrophages^60,61^. In the study by Nadra and colleagues, large mineral particles (>10 μm) failed to activate immune cells, indicating that immune cells activation may depend on physical constraints related to phagocytosis^61^. It is worth noting that other particle-related parameters, such as composition, crystallinity, and shape^10,60,62–64^, may also affect particle-neutrophil interactions, in addition to particle size. For instance, mineral and polymer microparticles with an irregular surface activate inflammasomes and recruit neutrophils more efficiently than particles with a smooth surface^64^. Taken together, our results suggest that large crystalline mineral aggregates of a few micrometers in diameter can elicit pro-inflammatory reactions, possibly as a result of their size, morphology and composition. These findings are consistent with our previous study on the interactions between mineralo-organic particles and macrophages^10^.

We observed that neutrophils externalize DNA in a ROS-dependent manner within 2 hours of exposure to mineralo-organic particles. Other pro-inflammatory molecules including PMA and pyocyanin also induce NETosis via a mechanism involving ROS^28,65^. The activation of NADPH oxidase and ROS production observed in particle-induced NETosis therefore appears to share similarities with the response of neutrophils to other stimuli such as microbial pathogens or PMA^28,33,65^. On the other hand, the involvement of NADPH oxidase in NET release is still controversial, since some studies have described ROS-independent NET release^66,67^. Altered mitochondrial functions may also be a feature of NETosis, although the loss of the mitochondrial membrane potential may not be sufficient to induce it^29^.

Neutrophil-macrophage interactions were previously reported^68^. Formation of DNA-spreading NETs can induce immune responses in other cells, including macrophages^69,70^. For example, NETs enhance IL-1β and IL-8 secretion by human platelets and macrophages as well as TNF-α release by macrophages^69^. Thus, communication via NETs may allow activation of bystander cells that are not in cell-to-cell contact with neutrophils. In short, NETs may serve as a bridge between neutrophils and macrophages during an immune response induced by stimuli such as mineral particles.

We have identified HMGB1 associated with NETs and showed its role in activating macrophages. The NET-mediated transmission of HMGB1 provides a novel mechanism for propagation of danger signals, since soluble HMGB1 is known to be released actively or passively by cells during necrosis. In this context, HMGB1 activates other cells such as dendritic cells and macrophages, leading to the production of IFN-α and TNF-α^49^. Although it is also possible that free HMGB1 is secreted by neutrophils upon contact with mineral particles or by activated macrophages, we showed that the amount of free HMGB1 released under these conditions was not sufficient to significantly induce TNF-α secretion by macrophages. Consistent with the *in vitro* findings, NETosis and NET extrusion were induced by stimuli of particles in the peritoneum of the C57BL/6 mice, and HMGB1 was required for the down-stream pro-inflammatory activity initiated by the particles. Given the relative scarcity of endogenous macrophages in the peritoneal environment within the short time frame studied, concurrent introduction of exogenous RAW264.7 cells was necessary as an amplifier of any response that might result from the intraperitoneal challenge. Since free-form HMGB1 is not known to be present until 8 hours post-stimuli^71^, the observed effect was likely due to the direct contribution of NET-bound HMGB1. These *in vivo* findings therefore provide the supporting evidence on the critical role of HMGB1-presenting NET in the innate response to the challenge of mineralo-organic particles.

TLR2 and TLR4, but not RAGE, have been previously implicated in HMGB1 signaling via activation of NF-κB *in vitro*, in both cell lines and primary cells^51,53,54^. In fact, TLR4 depletion attenuates HMGB1-mediated induction of IL-8 release and TNF-α production^54^. In contrast, RAGE appears to play a minor role in macrophage activation by HMGB1^51,53^. Consistent with these findings, we identified TLR2 and TLR4 as the targets of NET-bound HMGB1, whereas RAGE was dispensable for HMGB1-mediated macrophage activation.

Depending on the cysteine residue redox state, the HMGB1 molecule may have chemoattractant or cytokine-inducing activity, or neither^72,73^. Understanding redox regulation of particle-triggered, NET-bound HMGB1 may provide insights into HMGB1 activity during NET presentation and macrophage activation.

In conclusion, the findings presented here support the hypothesis that biological mineralo-organic particles elicit pro-inflammatory responses involving interaction between neutrophils and macrophages and paracrine-like delivery of danger signals that activate macrophages via NETs. Our findings demonstrate coordination of macrophage and neutrophil activity by NET-presented HMGB1 in response to pro-inflammatory stimuli. Elucidation of the role of HMGB1 signaling within the pro-inflammatory cascade represents a new advancementin  our understanding of host immunity, with implications for the understanding of diseases involving ectopic calcification as well as their prevention and treatment.

## Methods

### Preparation of mineralo-organic particles

FBS (Biological Industries) for particle preparation was heat-inactivated and then filtered through 0.2-μm disc filters. Particles were prepared by mixing CaCl_2_ and Na_2_HPO_4_ at a final concentration of 0.7 or 3 mM each in DMEM, pH 7.4, with 0.1% v/v FBS, 1.2 mg/ml BSA (Sigma), 0.12 mg/ml or 4–40 mg/ml BSF (Sigma). Sample incubation at 37 °C, 5% CO_2_, and saturated humidity was carried out for 3 days. Mineral particles were sized and counted by dynamic light scattering (DLS). Mixing 0.1% FBS or 1.2 mg/ml BSA with 3 mM each of CaCl_2_ and Na_2_HPO_4_ yielded micrometer-sized and crystalline particles. Lowering concentrations of both CaCl_2_ and Na_2_HPO_4_ to 0.7 mM while raising BSF to 40 mg/ml yielded nanometer-sized particles. Intermediate CaCl_2_, Na_2_HPO_4_, and BSF concentrations resulted in sub-micrometer particles (Table 1). Unless indicated otherwise, particles prepared by mixing calcium and phosphate (final concentration of 3 mM each) in DMEM with 0.1% v/v FBS were used in the present study.

In some microscopy experiments, mineralo-organic particles were labeled with FITC-BSA (Sigma) during preparation by adding FITC-BSA to the formation mixture. The mixture was then centrifuged at 13,000 × *g* for 1 min to harvest the pellet of FITC-BSA-labeled particles after removing supernatant. The pelleted particles were subject to washing to remove unbound FITC-BSA by repeated cycles of resuspension in fresh medium followed by centrifugation as described above.

### Dynamic light scattering

Characterization of particle sizes and numbers by DLS was described earlier^10^. Briefly, samples of particle suspension were transferred to disposable plastic cuvettes and mixed by gentle inversion prior to reading. Detection was performed using a Coulter N4 Plus submicron-particle size analyzer (Beckman Coulter) at 25 °C at an incident angle of 90 degrees.

### Transmission electron microscopy

TEM observation of particle structure was described^10^. Thin sections were prepared using a Leica Ultracut UCT microtome (Leica Microsystems). Specimens were observed without staining under a JEOL JEM-1230 TEM (JEOL, Tokyo, Japan) operated at 120 keV.

### Isolation of human neutrophils

Human blood was drawn from antecubital veins of healthy adult volunteers with no previously known disease, in accordance with the guidelines approved by the Institutional Review Board of Chang Gung Memorial Hospital (CGMH, Gueishan, Taiwan). Informed consents were obtained from all donors enrolled in the study. Neutrophils were isolated from whole blood by centrifuging the whole blood layered over Polymorphprep (Axis-Shield) at 500 × *g* for 35 min. The resultant stratum of polymorphonuclear cells was harvested and suspended in Dulbecco’s phosphate-buffered saline (DPBS) containing 0.1% w/v human serum albumin (HSA; Sigma). Erythrocytes were lysed using the ACK lysis buffer (0.15 M NH_4_Cl, 10 mM KHCO_3_, 0.1 mM Na_2_EDTA; pH 7.4), and 0.1% HSA/DPBS was used to resuspend the remaining cells post-lysis. Isolated neutrophils were then cultured in the presence or absence of mineral particles in FBS/DMEM on culture dishes coated with poly-D-lysine (BD Biosciences). Mouse anti-human CD177 antibody (BD Biosciences) was used to identify neutrophils. Mouse IgG isotype control (BD Biosciences) was used as the negative control. The flow cytometry profile of CD177-positive neutrophils (Supplementary Fig. S3) was comparable to the manufacturer’s specifications. In some experiments, neutrophils were pretreated with 50 μM GW311616A (Medchem Express), a neutrophil elastase inhibitor^74^, for 1 hour.

### Co-culture of neutrophils and macrophages

The concentration of particles was adjusted with DMEM prior to incubation with neutrophils. Neutrophils, either treated with particles for 2 hours at a constant particle-to-cell ratio or not, were co-incubated with THP-1, a human acute monocytic leukemia cell line (American Type Culture Collection; ATCC), either directly or in a Transwell insert for additional 8 hours. Neutrophils were separated from the macrophages in the lower well by a 0.4-μm pore size membrane (Corning). Prior to co-culture with neutrophils, THP-1 cells were cultured at 10^6^ cells/ml in RPMI 1640 medium containing 10% FBS. THP-1 cells were primed with 200 nM PMA (ENZO Life Science) for 24 hours. In some experiments, DNase I (Roche) was used to treat NETs. DNase I was added to culture at a final concentration of 10 units/ml. Recombinant human HMGB1 (PlexBio) was used to stimulate THP-1 for 8 hours at 100 ng/ml as a positive control. Chicken anti-HMGB1 polyclonal antibody was purchased from IBL international. Mouse anti-TLR2 and 4 and anti-RAGE-neutralizing antibodies were obtained from eBioscience and R&D Systems respectively, and used in some co-culture experiments at 10 μg/ml.

### Generation of human peripheral blood monocyte-derived macrophages

Peripheral blood mononuclear cell were isolated from blood drawn from healthy donors by centrifugation in Histopaque 1077 (Sigma) at 500 × *g* for 40 min and suspended in DPBS. The cells were washed and resuspended in RPMI 1640 containing 10% FBS. After 24 hours, unattached cells were removed, and the remaining cells were washed with DPBS and allowed to differentiate in RPMI 1640 with 100 ng/ml M-CSF for an additional 7 days into macrophages. Culture media was changed every two days. Macrophages were co-cultured with neutrophils, either treated with particles for 2 hours or not, for an additional 8 hours, in the presence or absence of chicken anti-HMGB1-neutralizing antibody.

### Stable shRNA expression by lentiviral transduction

THP-1 cells stably expressing shRNA against MyD88 (clone IDs TRCN0000008024, TRCN0000008025, TRCN0000008026, TRCN0000008027 and TRCN0000011223) and RAGE (clone IDs TRCN0000062658, TRCN0000062659, TRCN0000062660, TRCN0000062661 and TRCN0000062662) were generated by transducing the cells with lentiviral particles (Sigma). The shRNA sequence 5′-TTGGCAACCGCTTTTTG-3′, which targets firefly luciferase mRNA, served as the negative control. Infection of cells with lentiviral particles were performed following the manufacturer’s instructions. Briefly, 2 ml of THP-1 cell suspension at10^5^ cells/ml was transferred into a sterile 15-ml conical tube. Lentiviral particles in suspension were then added to THP-1 cells at a multiplicity of infection (MOI) of 5 in the presence of 8 μg/ml of polybrene. Incubation mixtures of cells and viruses were centrifuged at 800 × *g* for 30 min at 32 °C in the same 15-ml conical tube. Each cell pellet was resuspended in 2 ml of cultured medium and incubated at 37 °C for 24 hours. Culture supernatants were removed and replaced with 2 ml of complete RPMI 1640 with 10% v/v FBS containing 0.8 μg/ml puromycin (Invivogen). The puromycin-selection culture lasted 1 week. Clones that produced the highest level of protein downregulation were selected for further experiments (MyD88: Clone ID TRCN0000008025; RAGE: Clone ID TRCN0000062658) (Supplementary Fig. S4).

### Enzyme-linked immunosorbent assay

ELISA for detection of TNF-α in culture supernatant was performed based on the manufacturer’s instructions (R&D Systems). Changes in the level of free TNF-α in the presence of NADPH oxidase inhibitors including 50 μM APDC (Santa Cruz Biotechnology), 100 μM DPI (Enzo Life Sciences), and 500 μM APO (Chromadex) were assessed by ELISA to elucidate the role of ROS in response to particle stimuli. ELISA kit for HMGB1was purchased from Uscn Life Science.

### Confocal microscopy and live cell imaging

Poly-D-lysine-coated μ-dish (Ibidi GmbH) was loaded with neutrophils at 37 °C for 30 min. The plated neutrophils were then incubated with 5 μg/ml Hoechst 33342, 500 nM Sytox green and/or 1 μM TMRM (tetramethylrhodamine methyl ester; Life Technologies). THP-1 cells were incubated with CellMask orange according to manufacturer’s specifications (Life Technologies). Cells were monitored by multiphoton laser-scanning microscopy LSM 510 META NLO (Carl Zeiss) for recording or image photography at 37 °C with a heating unit XL S and TempModule S (Carl Zeiss). Each run of live cell monitoring was up to 3 hours long at the frame rate of 1 to 5 min per frame. Imaging analysis and reconstitution was performed with ZEN 2012 (Carl Zeiss). Percentage of neutrophils forming NETs was calculated by dividing the number of Sytox-positive cells with the total number of cells in each field of views 2 hours after addition of particles. For observation of HMGB1 signals, neutrophils were fixed with 4% paraformaldehyde for 10 min, followed by blocking with blocking buffer containing 5% FBS for 20 min. Fixed and blocked neutrophils were incubated with rabbit anti-HMGB1 antibody (Cell Signaling Technology) in blocking buffer for 1 hour before incubation with FITC goat anti-rabbit IgG secondary antibody (Jackson ImmnoResearch) for 45 min. Confocal microscopy was performed on stained cells using the LSM 780 microscope (Carl Zeiss). For observation of mice intraperitoneal cell population, images were taken using an Eclipse Ti fluorescence microscope (Nikon) with a stage top incubator set at 37 °C (Tokai Hit).

### Flow cytometry

For ROS detection, neutrophils incubated with particles for 1 hr were treated with CM-H_2_DCFDA (Life Technologies) and incubated for 10 min. The labeled cells were washed and subjected to flow cytometry using the FACSCalibur flow cytometer (Becton Dickinson). Cellquest Pro was used as the software for post-hoc analysis.

### Immunoblotting

For intracellular cytokine detection, neutrophils or THP-1 cells were collected by scraping and washed by centrifugation at 180 × *g* for 5 min at 4 °C in PBS. Cell pellets were resuspended in sodium dodecyl sulfate (SDS) loading buffer (20% glycerol, 3% SDS, 3% 2-mercaptoethanol, 10 mM Tris, 0.2% bromophenol blue; pH 6.8). Samples were boiled for 5 min and loaded onto a SDS-PAGE gel for electrophoresis. Separated protein were then transferred to PVDF membranes (Millipore) by tank electroblotting. Rabbit anti-TNF-α (Cell Signaling Technology) and mouse anti-β-actin (Millipore) antibodies were used for immunoblotting detection. For detection of HMGB1, 2.5 × 10^6^ cells were incubated for 4 hours with or without mineral particles. The cells were subsequently subjected to 1 hour of DNase treatment in medium containing 10 units/ml of DNase I and protease inhibitor cocktail (Thermo Scientific). The samples were centrifuged at 300 × *g* to remove cells and then at 13,000 × *g* to remove insoluble matters. 80% (v/v) ice-cold acetone was added to the supernatant, and the acetone-treated supernatant sample was incubated for 24 hrs at −20 °C. Protein pellets were obtained by centrifugation at 13,000 × *g* for 30 min at 4 °C. Pellets were air-dried and resuspended in SDS loading buffer for subsequent SDS-PAGE and immunoblotting analysis as described above. Rabbit anti-HMGB1 antibody (Cell Signaling Technology) was used to for detection. The secondary antibodies goat anti-mouse IgG (Jackson ImmunoResearch) and goat anti-rabbit IgG (Millipore) were used according to specifications from the supplier.

### Animal experiments

All procedures of husbandry and animal experiments were approved by the Institutional Animal Care and Use Committee (IACUC) of CGU in accordance with relevant guidelines and regulations governing animal use and welfare. Male and female wildtype C57BL/6N mice aged between 6 and 8 weeks were commercially obtained from BioLASCO (Taiwan). Mice were maintained in a 12-hour light/12-hour dark cycle at a temperature of 20 ± 2 °C with relative humidity at approximately 60% under the SPF facility of CGU. Food and water were provided ad libitum. Animals were intraperitoneally injected with mineralo-organic particles prepared as described in sections above or injected with the medium alone. Post-injection peritoneal cells were harvested by intraperitoneal lavage of 3 ml of PBS, pelleted by centrifugation at 400 × *g* for 10 min, and treated with ACK lysis buffer to remove the erythrocytes in the pellet. Erythrocyte-free peritoneal cells were then placed on culture dishes coated with poly-D-lysine and stained with Hoechst 33342 and Sytox, and examined by fluorescence microscopy (the Eclipse Ti fluorescence microscope; Nikon) at 37 °C with a stage top incubator (Tokai Hit).

RAW264.7 cells (ATCC) were pre-treated with 10 ng/ml IFN-γ for 16 to 18 hours in RPMI-complete medium. Two hours prior to introduction of the cells into peritoneum, mineralo-organic particles were intraperitoneally injected at 10 mg particles into C57BL/6 mice. Depending on the experimental design, the injection was carried out alone, with the addition of chicken IgY (IBL international) as isotype control or chicken anti-HMGB1-neutralizing Ab (both at 100 μg in 100 μl per animal), or with GW311616 (100 μg per injection). Particle-free injection with vehicle solution (DMEM + 0.1% FBS + DMSO) or LPS (10 μg/ml) was also performed as controls. For immune response boosting, interferon (IFN)-γ-primed RAW264.7 cells at 5 × 10^6^ cells/ml in DMEM complete media were used. Six hours after intraperitoneal particle challenge, the peritoneal lavage was harvested for ELISA assay of TNF-α, which was performed by injection of 1 ml of ice-cold PBS followed by gentle massage. The resultant peritoneal flush was then drawn out of the peritoneum and centrifuged (600 × *g* for 5 min at 4 °C).

### Statistical analysis

Statistical analysis was performed using unpaired t-test for comparisons of two groups. For comparison of three or more groups, one-way ANOVA was used, followed by Dunnett’s least significant difference test.

## Electronic supplementary material

Supplementary Information

Video S1

Video S2
