# Supplementary material for: Mineral particles stimulate innate immunity through neutrophil extracellular traps containing HMGB1
Source: Sci Rep. 2017 Nov 30;7:16628. doi: 10.1038/s41598-017-16778-4 (PMC5709501; doi:10.1038/s41598-017-16778-4)
Supplement: Supplementary file 1 — Supplementary Information [file 41598_2017_16778_MOESM1_ESM.pdf]

Mineral particles stimulate innate immunity through neutrophil extracellular traps containing HMGB1

Hsin-Hsin Peng, Yu-Ju Liu, David M. Ojcius, Chiou-Mei Lee, Ren-Hao Chen, Pei-Rong Huang, Jan Martel & John D. Young

Supplementary Information

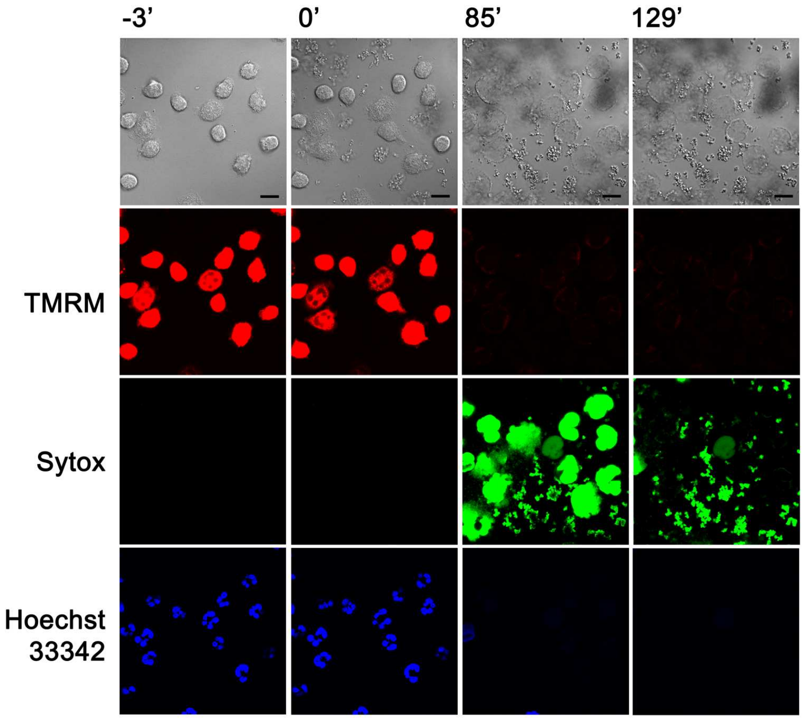

Figure S1. Mineral particles induce loss of mitochondrial potential in neutrophils. Neutrophils were exposed to mineral particles and an indicator of mitochondrial membrane potential (tetramethylrhodamine methyl ester, TMRM; red). Nuclear DNA was stained blue (Hoechst 33342), while NET DNA was stained green (Sytox). Data are representative of three independent experiments. Scale bar, 10 µm.

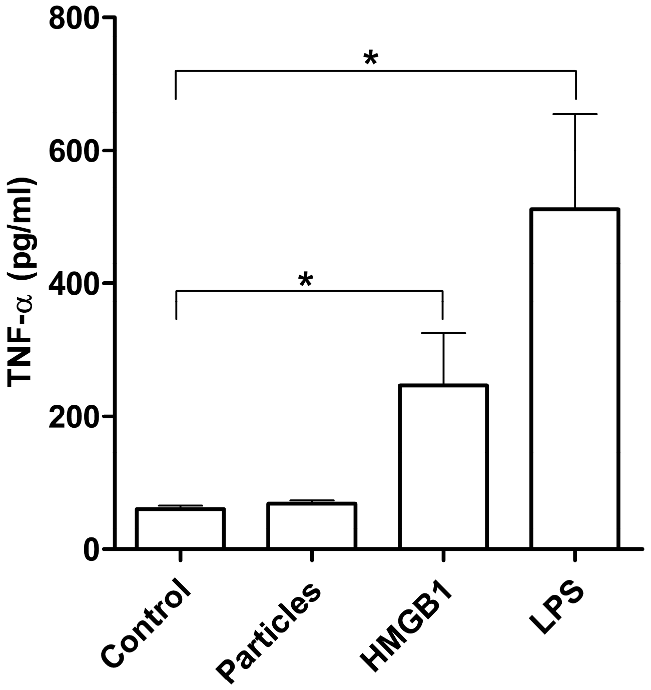

Figure S2. Priming THP-1 macrophages with PMA does not facilitate TNF-α secretion upon stimulation with mineral particles. THP-1 cells previously primed with PMA were cultured with mineralo-organic particles (“Particles”), or recombinant HMGB1 (“HMGB1”) or LPS (“LPS”) as positive control. Data are shown as means ± SEM and the results of at least three independent experiments. *$p < 0.05$, vs. untreated neutrophils.

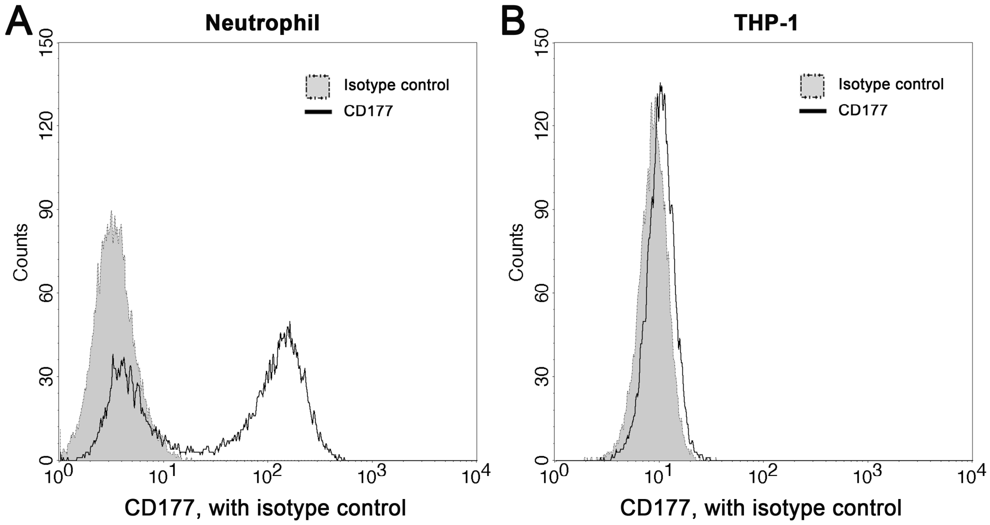

Figure S3. Flow cytometry analysis of isolated neutrophils and cultured macrophages. The isolated neutrophil population (A) stained positive for CD177, whereas cultured THP-1 cells (B) did not. A portion of neutrophils remained CD177 negative; the CD177-negative population is considered part of the neutrophil profile of CD77 expression.

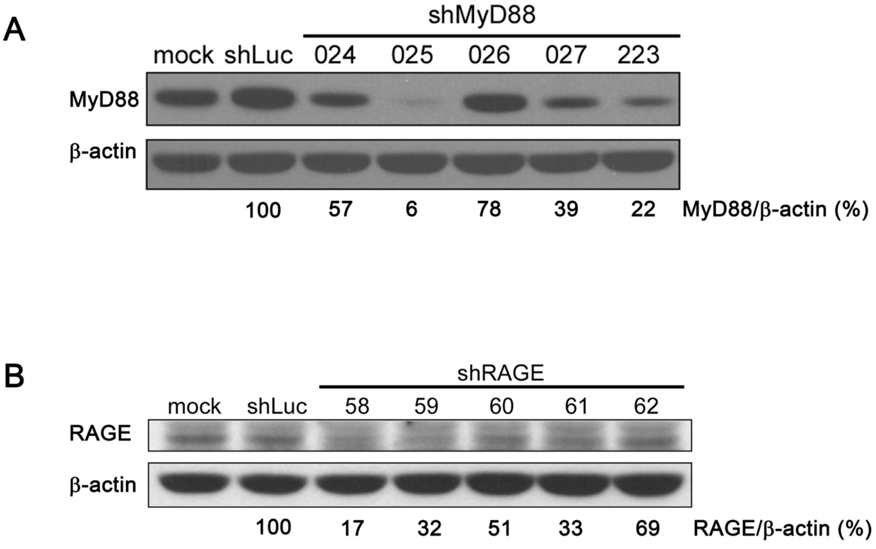

Figure S4. Downregulation of MyD88 and RAGE by stable shRNA expression in THP-1 cells. THP-1 cell clones stably expressing shRNA targeting either MyD88 (A) or RAGE (B) were generated, and expression of the target proteins was assessed using Western blotting. Mock: mock-transduced cells; shLuc: THP-1 cells expressing shRNA directed against luciferase as a control; 024, 025, 026, 027, and 233: MyD88-targeting shRNA clone TRCN0000008024, TRCN0000008025, TRCN0000008026, TRCN0000008027 and TRCN0000011223, respectively; 58, 59, 60, 61, and 62: RAGE-targeting shRNA clone TRCN0000062658, TRCN0000062659, TRCN0000062660, TRCN0000062661 and TRCN0000062662, respectively.

Video S1. Live cell imaging of neutrophil response to mineral particles

Microscopy images were taken every 3 min. The playback speed is 5 frames/sec. Blue, DAPI; green, Sytox.

Video S2. Depiction of 3D-reconstruction of NET-macrophage interactions

The movie was generated by Z-stacking with section thickness = 1 μm. Blue, DAPI; green, Sytox; red, THP-1.
